# Antitumor Effect of Sugar-Modified Cytosine Nucleosides on Growth of Adult T-Cell Leukemia Cells in Mice

**DOI:** 10.3390/vaccines8040658

**Published:** 2020-11-05

**Authors:** Naoyoshi Maeda, Akira Matsuda, Satoko Otsuguro, Masahiko Takahashi, Masahiro Fujii, Katsumi Maenaka

**Affiliations:** 1 Center for Research and Education on Drug Discovery, Faculty of Pharmaceutical Sciences, Hokkaido University, Kita-12, Nishi-6, Kita-ku, Sapporo 060-0812, Japan; matuda@pharm.hokudai.ac.jp (A.M.); otsuguro@pharm.hokudai.ac.jp (S.O.); 2 Division of Virology, Niigata University Graduate School of Medical and Dental Sciences, 1-757 Asahimachi-Dori, Niigata, Niigata 951-8510, Japan; masahiko@med.niigata-u.ac.jp (M.T.); fujiimas@med.niigata-u.ac.jp (M.F.); 3 Laboratory of Biomolecular Science, Faculty of Pharmaceutical Sciences, Hokkaido University, Kita-12, Nishi-6, Kita-ku, Sapporo 060-0812, Japan

**Keywords:** adult T-cell leukemia, cytarazid, DMDC, FDMDC, NOG mice

## Abstract

Adult T-cell leukemia (ATL) is a CD4^+^ T-cell neoplasm caused by human T-cell leukemia virus type I. As the prognosis for patients with ATL remains extremely poor due to resistance to conventional chemotherapy regimens, introduction of novel therapeutic agents is needed. Previous studies have reported that nucleosides 2′-deoxy-2′-methylidenecytidine (DMDC) and its derivative 2′-deoxy-2′-methylidene-5-fluorocytidine (FDMDC) exhibit antitumor activities in T-cell acute lymphoblastic leukemia (T-ALL) and solid tumor cell lines. Another nucleoside, 1-(2-azido-2-deoxy-β-D-arabinofuranosyl)cytosine (cytarazid), is considered a therapeutic drug with antitumor activity in human solid tumors. In this study, we investigated the effects of these nucleosides on cell growth in vitro and in vivo using relevant leukemia cell lines and NOD/Shi-*scid*, *IL-2Rg^null^* (NOG) mice, respectively. The nucleosides demonstrated significant cytotoxic effects in ATL and T-ALL cell lines. Intraperitoneal administration of FDMDC and DMDC into tumor-bearing NOG mice resulted in significant suppression of tumor growth without lethal side effects. Our findings support a therapeutic application of these nucleosides against tumor progression by targeting DNA polymerase-dependent DNA synthesis in patients with ATL.

## 1. Introduction

Adult T-cell leukemia (ATL) is a highly aggressive CD4^+^ T-cell leukemia characterized by clonal integration of the human T-cell leukemia virus type I (HTLV-I) in tumor cells [1]. Aggressive ATL is classified into acute, lymphoma, and unfavorable chronic types. Currently, the recommended first-line treatment for patients with aggressive ATL is a multiagent combination chemotherapy consisting of VCAP (vincristine, cyclophosphamide, doxorubicin, and prednisolone), AMP (doxorubicin, ranimustine, and prednisolone), and VECP (vindesine, etoposide, carboplatin, and prednisolone) with granulocyte colony-stimulating factor [2]. Nevertheless, the prognosis for patients with ATL remains extremely poor due to resistance to conventional chemotherapy regimens. Specifically, mean survival times for patients with acute, lymphoma, and unfavorable chronic types are approximately 6.2, 10.2, and 15 months, respectively [2]. Depending on the responsiveness to chemotherapy, age, and availability of human leukocyte antigen-matched donors, allogeneic hematopoietic stem cell transplantation (allo-HSCT) may be recommended [3]. While multiagent chemotherapy and allo-HSCT improve prognosis, further development of novel therapeutic drugs and regimens is urgently required.

Recently, a variety of chemical compounds and pharmacological inhibitors targeting the tumor itself based on different molecular mechanisms have been evaluated in clinical trials, including enhancer of zeste homolog 2, histone deacetylase, and checkpoint inhibitors [3]. Among the various chemical agents used in tumor chemotherapy, antitumor nucleosides have been developed for use in humans [4]. The nucleoside analog 2′,2′-difluoro-2′-deoxycytidine, also known as gemcitabine, has been approved and is widely used for patients with pancreatic cancer and non-small-cell lung cancer (NSCLC) [5,6]. Another deoxycytidine analog, 1-β-D-arabinofuranosylcytosine (cytarabine, also known as cytosine arabinoside or ara-C), exhibits antitumor activity in the treatment of acute myeloid leukemia and Hodgkin’s lymphoma [7,8]. Similar in structure to cytarabine, 2′-deoxy-2′-methylidenecytidine (DMDC) has been shown to have a distinct mechanism of tumor inhibition [4]. Specifically, DMDC is activated by deoxycytidine kinase (DCK) to its 5′-monophosphate, which is further phosphorylated to 5′-diphosphate and triphosphate metabolites; the resultant DMDC diphosphate inhibits ribonucleotide reductase, which is involved in DNA synthesis, and the DMDC 5′-triphosphate inhibits elongation activity of DNA polymerase by incorporating into genomic DNA [4]. Thus, these two nucleotide metabolites work in combination to induce DNA strand breaks, resulting in apoptosis [4]. Given the proposed molecular mechanisms, DMDC shows much higher antitumor effects on human leukemic cells such as T-cell acute lymphoblastic leukemia (T-ALL) cell lines than on solid tumors in vitro [9]. In addition, DMDC exhibits antitumor activities in human tumors in vivo [10,11]. Due to favorable pharmacokinetic and clinical parameters analyzed in vitro and in vivo [12,13,14,15], phase I clinical trials using DMDC were performed in Japan [16]. A derivative of DMDC, 2′-deoxy-2′-methylidene-5-fluorocytidine (FDMDC) also shows antitumor activities on the above tumor cell lines in vitro [9] but has not been tested in vivo. Another nucleoside, 1-(2-azido-2-deoxy-β-D-arabinofuranosyl)cytosine (cytarazid), its 5′-triphosphate inhibits α- and β-DNA polymerase and is considered a therapeutic drug with antitumor activity in human solid tumors [17].

In this study, we examined the antitumor effects of DMDC, its derivative FDMDC, and cytarazid on the growth of ATL cells. All nucleosides induced significant cytotoxic effects in ATL cell lines in a dose-dependent manner in vitro. We further examined the antitumor effects of DMDC and FDMDC using NOD/Shi-*scid*, *IL-2Rg^null^* (NOG) mice, which are widely utilized for evaluating drug efficacy in ATL and other human malignant tumors. Treatment of ATL tumor-bearing NOG mice with DMDC and FDMDC resulted in significant inhibition of tumor growth in vivo. Notably, this is the first report of the antitumor activity of FDMDC in a human tumor xenograft model in vivo. Overall, our results support the potential use of nucleosides as a therapeutic strategy for patients with ATL.

## 2. Materials and Methods

### 2.1. Cells

The HTLV-I-transformed T cell lines C5/MJ, HUT-102, MT-2, MT-4, and SLB-1; ATL-derived (HTLV-I-positive) cell lines KOB, MT-1, ST1, and TL-OmI; and T-ALL (HTLV-I-negative) cell lines CCRF-CEM and MOLT-4 were grown in Roswell Park Memorial Institute (RPMI)-1640 medium supplemented with 10% heat-inactivated fetal bovine serum (FBS), penicillin (100 units/mL), and streptomycin (100 µm/mL). Recombinant human interleukin (IL)-2 (0.5 nM) (PeproTech, NJ, USA) was added to the culture of KOB and ST1. The Burkitt lymphoma cell lines BJAB and Raji were grown in RPMI-1640 medium supplemented with 20% FBS, penicillin, and streptomycin. The colon adenocarcinoma cell line SW480 was grown in Dulbecco’s modified Eagle’s medium (D-MEM) supplemented with 10% FBS, penicillin, and streptomycin.

### 2.2. Nucleoside Synthesis

**Figure 1:**
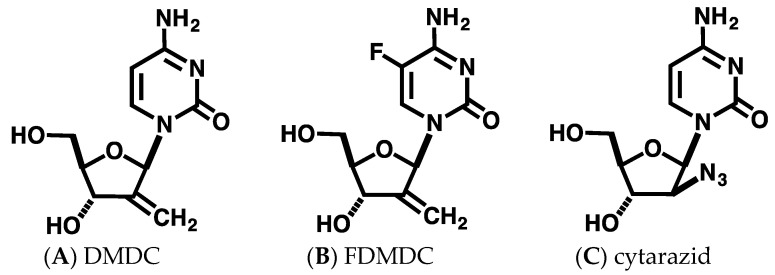
Chemical structure of nucleosides used in this study. Structures of DMDC (**A**), FDMDC (**B**), and cytarazid (**C**) are shown.

DMDC, FDMDC, and cytarazid (Figure 1A–C) were synthesized as previously described [9,17]. The nucleosides were dissolved in dimethyl sulfoxide (DMSO) to a final concentration of 100 mg/mL.

### 2.3. In Vitro Cell Proliferation Assay

Cells were seeded at a density of 2 × 10^4^ cells in the presence of various concentrations of nucleosides for 72 h at 37 °C. DMSO was used as the control vehicle. The cells were further incubated with the Cell Proliferation Reagent WST-1 (Roche, Mannheim, Germany) for 2–4 h at 37 °C. Absorbance of the samples was measured at 450 and 550 nm (reference wavelength) using an EnSpire microplate reader (PerkinElmer, MA, USA).

### 2.4. Mice

NOG mice were obtained from the Central Institute for Experimental Animals (Kawasaki, Japan). All mice were maintained under specific-pathogen-free conditions in the Laboratory of Animal Experiments, Hokkaido University.

### 2.5. Tumor Xenograft Model

TL-OmI cells were washed twice with serum-free RPMI-1640 medium. The cells were resuspended in serum-free RPMI-1640 medium and inoculated subcutaneously into NOG mice at a density of 3 × 10^7^ cells per mouse. For therapeutic experiments, 50 or 100 mg/kg of FDMDC, DMDC, or DMSO as a control vehicle, resuspended in phosphate-buffered saline (−), was intraperitoneally injected into NOG mice once a day for 5 days when tumors reached approximately 150–250 mm^3^ after cell inoculation. Tumor size was measured daily and determined using the formula (L × W^2^)/2 (L, length; W, width). Body weight was measured daily. All experiments were approved and performed in accordance with the guidelines of the Committee of Ethics on Animal Experiments at Hokkaido University.

### 2.6. Statistical Analysis

Statistically significant differences between nucleoside- and DMSO-treated mice were calculated using Student’s *t*-test and are indicated as *p* values. Differences of *p* < 0.05 were considered statistically significant (*, *p* < 0.05; **, *p* < 0.01).

## 3. Results

### 3.1. Effects of Nucleosides on Growth of ATL Cell Lines In Vitro

**Figure 2:**
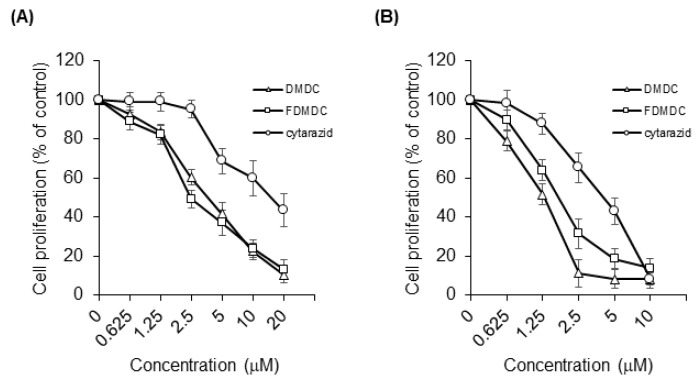
DMDC, FDMDC, and cytarazid inhibited cell growth of ATL cell lines and HTLV-I-transformed cell lines in vitro. TL-OmI cell lines (**A**) and MT-2 cell lines (**B**) were seeded at a density of 2 × 10^4^ cells in the presence of various concentrations of nucleosides DMDC, FDMDC, and cytarazid for 72 h at 37 °C. The cells were further incubated with the Cell Proliferation Reagent WST-1 for 2–4 h at 37 °C. For (**A**) and (**B**), bars indicate mean values ± standard error of the mean (n = 3).

Previous studies have reported the effects of DMDC and its derivative FDMDC (Figure 1A,B) on leukemia cells and solid tumors in vitro, including strong cytotoxicity to T-ALL cell lines [9]. Thus, we hypothesized that these compounds would also induce death in ATL cells. First, we performed a WST-1 cell proliferation assay to examine the antitumor effect of DMDC, and FDMDC; we found that they suppressed the viability of various tumor cell lines in a dose-dependent manner (representative examples are shown in Figure 2A,B).

The half-maximal inhibitory concentration (IC_50_) values of DMDC, FDMDC, and cytarazid in tested cell lines are summarized in Table 1. We confirmed its effect in T-ALL cell lines CCRF-CEM and MOLT-4 (IC_50_ of ~0.6 µM for both) and colon adenocarcinoma cell line SW480 (IC_50_ of 10.1 µM) as control cell lines, in agreement with previous results [9]. We focused our analysis on four ATL cell lines and five HTLV-I-transformed cell lines. DMDC exhibited strong cytotoxicity in both the ATL cell lines (IC_50_ of 2.01 to 4.88 µM) and HTLV-I-transformed cell lines (IC_50_ of 1.49 to 2.36 μM). Similar to DMDC, FDMDC exhibited cytotoxic effects on the growth of ATL cell lines (IC_50_ of 1.53 to 3.86 µM) and HTLV-I-transformed cell lines (IC_50_ of 1.62 to 4.49 µM). We also tested two Burkitt lymphoma cell lines, BJAB and Raji; BJAB cells were highly sensitive to both DMDC and FDMDC (IC_50_ of ~0.05 µM for both). Finally, we tested the antitumor effect of cytarazid (Figure 1C); it also exhibited cytotoxicity in the cell lines described above for DMDC and FDMDC (Figure 2A,B). However, IC_50_ values of cytarazid in the four ATL cell lines and four HTLV-I-transformed cell lines (IC_50_ of 5.46 to 21.1 µM) were much higher than those of DMDC or FDMDC, with the exception for MT-4 cells (IC_50_ of 0.353 µM).

### 3.2. Treatment of ATL Tumor-Bearing NOG Mice with FDMDC and DMDC

**Figure 3:**
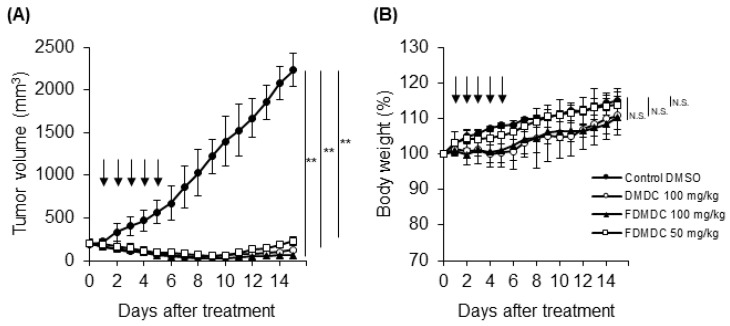
FDMDC and DMDC suppressed tumor growth of ATL cell lines inoculated into NOG mice in vivo. NOG mice were subcutaneously inoculated with 3 × 10^7^ TL-OmI cells. After tumor size reached approximately 150–250 mm^3^, the indicated doses of FDMDC (n = 8), DMDC (n = 8), or control DMSO (n = 8) were intraperitoneally administered into the mice daily for 5 days (as indicated by arrows). Tumor size (**A**) and body weight (**B**) were measured daily. For (**A**) and (**B**), bars indicate mean values ± standard error of the mean for the eight mice in each group obtained from among two independent experiments. Statistically significant differences are shown as *p* values (**; *p* < 0.01). N.S., no significant difference.

To evaluate the antitumor effects of the nucleosides on the growth of ATL cells in vivo, we investigated a xenograft model using NOG mice. The TL-OmI cell lines were subcutaneously inoculated into NOG mice. After tumor size reached approximately 150–250 mm^3^, we intraperitoneally administered DMDC and FDMDC at a dose of 50 or 100 mg/kg into TL-OmI tumor-bearing NOG mice once a day for 5 days, as previously described [10]. Intraperitoneal administration of DMDC at a dose of 100 mg/kg resulted in significant inhibition of tumor growth in TL-OmI tumor-bearing NOG mice (Figure 3A), in agreement with previous reports on human tumor xenografts [10,11]. Notably, administration of FDMDC at doses of 50 and 100 mg/kg also showed significant inhibition of ATL tumor growth (Figure 3A). DMDC and FDMDC were very effective on tumor development, and tumor volume drastically decreased (becoming almost invisible) day by day during treatment. Mice treated with FDMDC at a dose of 50 mg/kg did not show any loss of body weight (Figure 3B). Although mice treated with 100 mg/kg DMDC or FDMDC had slightly reduced body weight as compared to those treated with DMSO, differences among the groups were not statistically significant (Figure 3B). In addition, their body weights returned to normal after completion of treatment. Thus, we concluded that the dosages tested in this study were not lethal to ATL tumor-inoculated mice. Importantly, this is the first report demonstrating the antitumor effect of FDMDC on the growth of human tumor cells in vivo.

## 4. Discussion

In the present study, we assessed the antitumor effects of structurally similar nucleosides DMDC, FDMDC, and cytarazid (as shown in Figure 1) on the growth of ATL cells. All nucleosides demonstrated potent cytotoxicity in ATL cell lines in vitro, and FDMDC and DMDC exhibited antitumor effects in vivo. Our results suggest that these nucleosides may be promising agents for use in ATL chemotherapy, which is currently of limited effectiveness.

We observed that DMDC and FDMDC were cytotoxic in all ATL and HTLV-I-transformed cell lines tested, although their effects varied among cell lines, even in identical tumor types (e.g., DMDC in TL-OmI or FDMDC in MT-4). Miwa et al. reported the relationship between antitumor effects and cytidine deaminase (CDA) activity in solid tumor cell lines [11]; since DMDC is resistant to CDA, the antitumor effect of DMDC is dependent on its activity. The levels of CDA expression and activity in ATL cells and other leukemia and lymphoma cells are unknown. A3G, a member of the APOBEC3 family, is a CDA that is predominantly expressed in lymphoma cells and is involved in mutational double-strand DNA break repair; therefore, APOBEC-dependent mutational processes should be investigated [18]. Considering that CDA is involved in DNA repair and damage control mechanisms, protein levels of CDA and somatic mutations in ATL cells should be assessed to evaluate the clinical efficacy of nucleosides. In addition to CDA activity, Miwa et al. measured DCK activity [11]. As DCK phosphorylates DMDC to its 5′-monophosphate, which is then phosphorylated to active diphosphate and triphosphate metabolites that induce apoptosis, the first phosphorylation of DCK is a critical factor in the antitumor effects of DMDC. The IC_50_ values of DMDC in ATL cell lines were higher than those in T-ALL cell lines, suggesting that DCK activity or protein levels in ATL cell lines may be lower than those in T-ALL cell lines, which should be further investigated. While we performed the WST-1 assay to assess cell proliferation, we did not perform any experiments in this study investigating ATL cell death caused by these nucleosides in vitro. We believe that using other assays to investigate biological mechanisms of apoptosis may uncover new leads for identifying effective uses for these nucleosides.

In our in vivo study, we found significant antitumor activities of DMDC and FDMDC in suppressing ATL tumor growth in NOG mice. Administration of DMDC at a dose of 100 mg/kg resulted in a slight body weight loss in tumor-bearing mice (Figure 3B), suggesting that the concentration induced cytotoxicity by nonspecific incorporation into normal cells. Thus, methods for increasing tumor-specific targeting of DMDC in human patients should be considered. In a preliminary experiment, FDMDC administered intraperitoneally at a dose of 25 mg/kg in one mouse showed lower effects than at doses of 50 and 100 mg/kg (data not shown). Further investigation using larger groups at lower doses will aid in clarifying the dose–response relationship of these nucleosides. In addition, we did not gather data on tumor growth during the period between inoculation and the initiation of treatment; these limitations should be noted and improved in future experiments.

Phase I clinical trials demonstrated that the maximum tolerated dose of DMDC and the recommended dose for phase II clinical trials were both 18 mg/m^2^/day [16]. Although dose-limiting toxicities such as anorexia, leukopenia, and anemia were observed in the trials, daily oral administration of DMDC for 14 days repeated at 4-week intervals induced responses in all patients with NSCLC and colorectal cancer. DMDC also showed antitumor activity in one patient with advanced NSCLC. In our study, the IC_50_ of DMDC and FDMDC in ATL cell lines and HTLV-I-transformed cell lines had the same or greater level of cytotoxic effect as that in SW480 cells (Table 1), indicating that the schedule recommended above can be used for patients with ATL or other hematological malignancies in clinical trials.

## 5. Conclusions

In conclusion, our results demonstrated the antitumor effects of nucleosides DMDC, FDMDC, and cytarazid on ATL tumor growth in vitro and in vivo. The clinical efficacy of these nucleosides on primary ATL cells should be explored in future studies. This work may lead to a promising therapeutic strategy for eradicating tumors in patients with ATL.

## Figures and Tables

**Table 1:** Antitumor effects of DMDC, FDMDC, and cytarazid on the growth of various human tumor cell lines in vitro.

Origin	Cell lines	IC_50_ (µM) ^1^		
		DMDC	FDMDC	Cytarazid
ATL	KOB	2.01	1.53	6.92
	MT-1	3.19	3.28	18
	ST1	2.1	2.91	18.6
	TL-OmI	4.88	3.86	21.1
HTLV-I-transformed	C5/MJ	2.2	3.59	11.5
	HUT-102	2.06	3.79	13.7
	MT-2	1.49	1.62	5.46
	MT-4	2.36	4.49	0.353
	SLB-1	1.73	3.31	15.5
T-ALL	CCRF-CEM	0.611	0.834	1.95
	MOLT-4	0.645	0.578	2.19
Burkitt lymphoma	BJAB	0.0501	0.0497	0.169
	Raji	11.3	14.1	28.1
Colon adenocarcinoma	SW480	10.1	17.6	47.9

The IC_50_ values were determined from the means of triplicate data in three independent experiments, as shown in Figure 2.
